# Supplementary material for: Theory of optimal balance predicts and explains the amplitude and decay time of synaptic inhibition
Source: Nat Commun. 2017 Mar 10;8:14566. doi: 10.1038/ncomms14566 (PMC5353699; doi:10.1038/ncomms14566)
Supplement: Supplementary Information — Supplementary Figures, Supplementary Tables, Supplementary Results, Supplementary Methods and Supplementary References [file ncomms14566-s1.pdf]

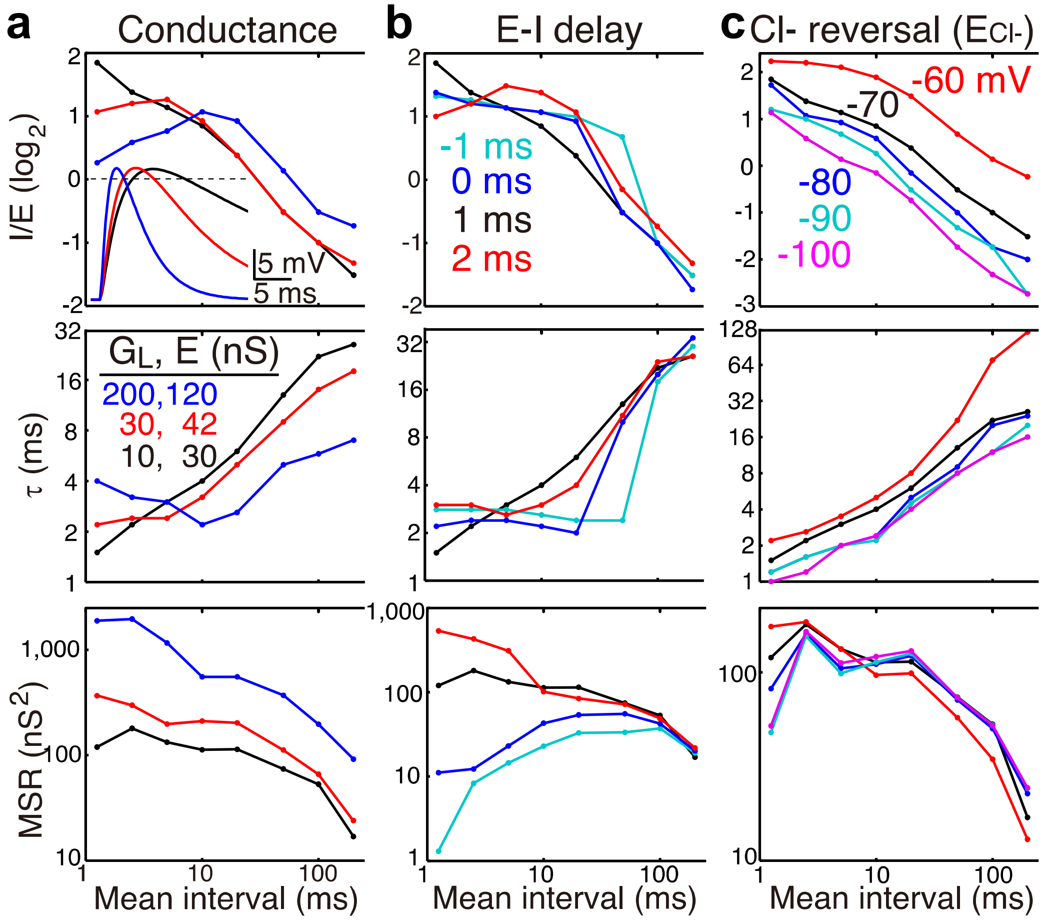

Supplementary Figure 1 Influence of conductance, E-I delay, and IPSG reversal potential on optimal I/E (top), τ (middle), and MSR (bottom) as a function of mean EPSG interval. This figure is analogous to figure 7 but for simulations without AP. **(a)** Membrane conductance was low (black; E=30 nS, G_L=10 nS), medium (red; E=42 nS, G_L=30 nS), and high (blue; E=120 nS, G_L=200 nS). The three combinations of E and G_L were chosen so that from resting membrane potential of -70 mV, an EPSG would cause an EPSP with peak near -48 mV in the absence of IPSG (inset at top). **(b)** E-I delays of -1 (cyan), 0 (blue), 1 (black) and 2 ms (red). **(c)** IPSG reversal potentials (E_{Cl-}) of -60 (red), -70 (black), -80 (blue), -90 (cyan), and -100 mV (magenta).

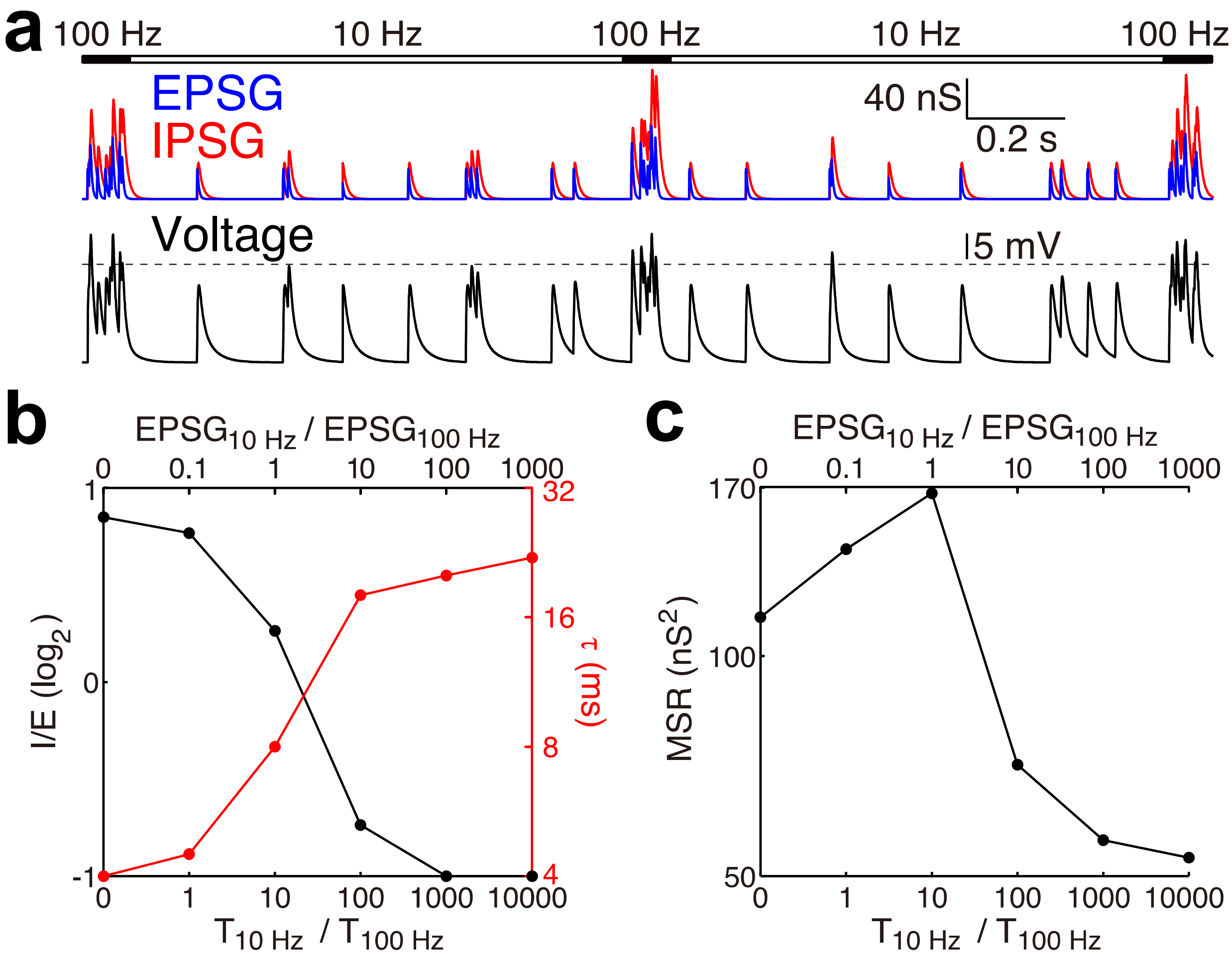

Supplementary Figure 2 Mixed high and low EPSG frequencies. As a first step toward considering more natural EPSG patterns, we identified optimal IPSG parameters for a neuron receiving alternating periods of EPSG at 10 and 100 Hz. The period of 100 Hz was fixed at 0.1 s per cycle, and we varied the period at 10 Hz from 0 to 1000 s. **(a)** Example of EPSG and optimal IPSG (top) and voltage (bottom) during 2.5 cycles in which the period of low frequency was 1.0 s, corresponding to 10-fold greater time spent at 10 Hz but equal expected numbers of EPSG at 10 and 100 Hz. **(b)** Optimal I/E and τ as a function of the ratio of time at 10 and 100 Hz (bottom axis) and the ratio of the expected number of EPSG at 10 and 100 Hz (top axis). When time at 10 Hz was 1000 s, optimal IPSG parameters were the same as those found with only 10 Hz (Fig. 5b). (c) Same as 'b' but showing MSR. When the numbers of EPSG at each frequency were equal, MSR was maximal.

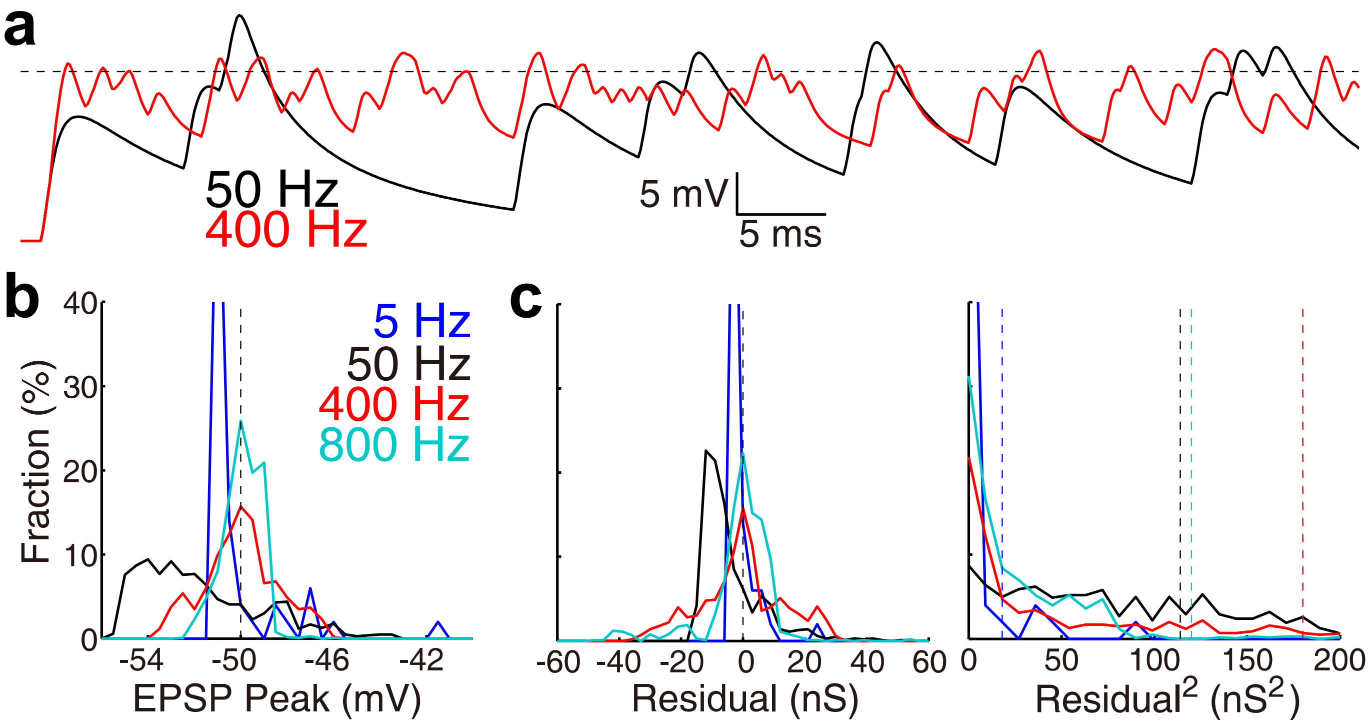

Supplementary Figure 3 Distributions of EPSP peaks and residuals are more symmetrical at higher frequencies. This explains the influence of EPSG frequency on spike probability (Fig. 9a). All data is from our standard model with optimal IPSG but without AP. **(a)** Examples of membrane voltage at 50 (black) and 400 Hz (red) at the start of simulations, which always began at the resting potential of -70 mV. **(b)** Distributions of EPSP peak voltage for 5 (blue), 50 (black), 400 (red) and 800 Hz (cyan). The maximum at 5 Hz is not shown but was 64%. **(c)** As in 'b' but showing distributions of residuals (left) and squared residuals (right). Dashed vertical lines indicate a residual of zero (left) and MSR (right). The sign of residuals (left) was chosen so that EPSP peaks that are positive of spike threshold (-50 mV) are associated with positive residuals. The maxima at 5 Hz are not shown but were 70% (left) and 84% (right). Squared residuals greater than 200 nS^2 are not shown.

	Neuron / Study	EPSG (Hz)	Species	Age (days)	T (℃)	τ (ms)	Notes
Monoaminergic & Basal Ganglia	**NA neurons (LC)**	**10**				**16.7**	
	Bruzos-cidon et al., 2015		Rats	175-250 g	36	15, 14.0	
	Jin et al., 2013		Mice	14-21	33.5	25, 19.3	mIPSC
	**DA neurons (SNc)**	**10**				**9.7**	
	Nugent et al., 2007		Rats	16-21	30	16.8, 10.0	τ from figure
	Matsui et al., 2014		Rats	150-300 g	35	10.8, 9.3	τ from figure
	**5-HT neurons (DR)**	**10**				**9.7**	
	Kirby et al., 2008		Rats	175-250 g	33	13.1, 9.7	τ from figure
	**ACh neurons (striatum)**	**10**				**11.0**	
	Matsui et al., 2014		Rats	150-300 g	35	13.7, 11.8	τ from figure
	Momiyama and Koga, 2001		Rats	12-20	23	28.8, 10.2	τ from figure
	**SN reticulata**	**200**				**1.8**	
	Miyazaki and Lacey, 1998		Rats	24-30	34	2.3, 1.8	
Cortical & Thalamic	**Dentate Granule cells**	**50**				**3.6**	
	Bartos et al., 2001		Rats	18-25	32	5.2, 3.6	
	**Dentate Basket cells**	**200**				**1.7**	
	Bartos et al., 2001		Rats	18-25	32	2.4, 1.7	
	**Visual Reticular (TRN)**	**24**				**20.7**	
	Huntsman and Huguenard, 2000		Rats	42-60	36	22.3, 20.7	sIPSC
	**Visual TC (LGN)**	**40**				**7.4**	
	Blitz and Regehr, 2005		Mice	26-32	38	8.0, 8.6	τ from figure
	Bright et al., 2007		Mice	≥30	36.5	6.4, 6.2	sIPSC
	**Somatosensory TC (VB)**	**40**				**7.0**	
	Huntsman and Huguenard, 2000		Rats	42-60	36	5.8, 5.4	sIPSC
	Wanaverbecq et al., 2008		Rats	21-28	34.5	10.4, 8.6	sIPSC
	**Auditory TC (MGv)**	**40**				**9.3**	
	Venkataraman and Bartlett, 2013		Rats	27	35	10.8, 9.3	
Cerebellar	**Inferior Olive**	**5**				**50.0**	
	Best and Regehr, 2009		Rats	23-25	34.5	60, 50.0	τ at 20 Hz (Fig. 4B)
	**DCN Nucleo-olivary**	**40**				**17.7**	
	Najac and Raman, 2015		Mice	20-30	33.5	23, 17.7	
	**Granule cells**	**50**				**7.0**	
	Crowley et al., 2009		Rats	19-40	33.5	9, 7.0	Estimated median sIPSC τ (Fig. 2J)
	**Purkinje cells**	**150**				**2.7**	
	Vincent et al., 1992		Rats	9-22	RT	7, 2.9	RT estimated at 25℃
	Houston et al., 2009		Rats	12	23	7, 2.5	
	**DCN Principal neurons**	**200**				**1.9**	
	Najac and Raman, 2015		Mice	20-30	33.5	2.0, 1.5	
	Person and Raman, 2012		Mice	13-29	36	2.4, 2.2	
Early Auditory	**VCN Bushy cells**	**100**				**12.4**	
	Xie and Manis, 2013b		Mice	20-35	34	10.4, 8.3	
	Nerlich et al., 2014		Gerbils	22-33	33	22, 16.4	
	**VCN T-Stellate cells**	**350**				**1.0**	
	Xie and Manis, 2013a		Mice	29-39	34	1.23, 1.0	
	**MNTB Principal neurons**	**125**				**2.1**	
	Awatramani et al., 2004		Rats	22-27	37	1.0, 1.0	
	Mayer et al., 2014		Gerbils	12-18	36.3	3.33, 3.2	
	**MSO Principal neurons**	**600**				**1.7**	
	Magnusson et al., 2005		Gerbils	17	36	2.0, 1.9	
	Smith et al., 2000		Rats	14	25	4, 1.6	
	Couchman et al., 2010		Gerbils	60-100	35	1.76, 1.5	
	**LSO Principal neurons**	**600**				**1.5**	
	Kramer et al., 2014		Mice	19	37	1.2, 1.2	
	Wu and Kelly, 1995		Mice	21-45	34	1.3, 1.0	
	Magnusson et al., 2005		Gerbils	17	36	1.99, 1.9	
	Sterenborg et al., 2010		Mice	11-15	25	4.6, 1.9	

Supplementary Table 1 Summary of experimental data on EPSG input rates and IPSG decay times used in figure 10a,b. The weighed decay time constant (τ) is given at the experimental temperature (left) and after

adjustment to 37º (right) based on a Q10 of 2.1. IPSG were evoked, except for neurons in which we were not able to find data on evoked IPSG and instead used miniature or spontaneous IPSG (see ‘notes’). Some studies did not report decay times but showed IPSC from which we calculated decay time (noted “τ from figure”). Rows in bold font provide estimates of EPSG input rate and the value of τ used in figure 10 (which is the mean across studies at 37º). References for EPSG input rates are discussed in Supplementary Methods.

Study	Brain Region	Species	Age (days)	E-I delay (ms)	I/E	Notes
Shen et al., 2011	Frog Visual Tectum	Xenopus	stage 48	3.94	0.85	Isolated brain, 21℃; E-I delay from Akerman and Cline (2006)
Blitz and Regehr, 2005	LGN Thalamocortical	Mice	26-32	1	1.1	in vitro, 38℃; Fig. 3B; opponent I/E 0.8
Chadderton et al., 2004	Cerebellar Granule	Rats	18-27		1.1	Anesthetized; tactile stimulus
Higley and Contreras, 2006	S1 L4	Rats	350-450 g	0.5	2.69	Anesthetized; fast PW deflection
Heiss et al., 2008	S1 L4	Rats	28-35	1.2	1.8	Anesthetized; PW at 10 mm/s; Fig. 2F, I/E 0.5 after adaptation;
Liu et al., 2010	V1 L2/3	Mice	260-480	5	1.48	Anesthetized; checkerboard, 4-5º, 70% contrast; Fig. 4e
Haider et al., 2013	V1 L2/3	Mice	120-180		3.2	Awake; vertical bars of 9º at 100% contrast
Wehr and Zador, 2003	A1	Rats	17-24	2.4	0.74	Anesthetized; 6-66 dB
Froemke et al., 2007	A1	Rats	90-150		1.3	Anesthetized; 60-80 dB; Fig. S4
Dorrn et al., 2010	A1	Rats	25-30	2.6	1.27	Anesthetized
Sun et al., 2010	A1 L4	Rats	≥80	1.7	1.28	Anesthetized; 70 dB; Fig. 3e; E-I delay Fig. 3f
Zhou et al., 2010	A1 L6, normal type	Rats	90	1.58	0.5	Anesthetized; 70 dB; Fig. 3e; E-I delay Fig. 3f
	A1 L6, silent type	Rats	90	-1.62	3.0	
Zhou et al., 2014	A1 L2/3	Mice	150-210	1.95	2.60	Awake; 70 dB
	A1 L4	Mice	150-210	1.95	2.33	
				1.85±1.59	1.68±0.84	mean±sd

Supplementary Table 2 Summary of experimental data on I/E used in figure 10c. All neurons in cerebral cortex were 'regular spiking.' Values of I/E were obtained from figures in some studies, as noted. All data in auditory neurons was obtained in response to pure tones at the neuron's characteristic frequency. The two studies in S1 barrel cortex measured E-I delays and I/E before and after adaptation to repeated deflection of the principle whisker (PW) at 10-18 Hz. Data presented here is 'before adaptation.' E-I delay was 1-2 ms greater after adaptation. I/E declined during adaptation in one study, as noted. For Haider et al (2013), I/E was measured from "center" in their Fig. 9c. Like all other values of I/E reported here, it was an arithmetic mean across cells (not the geometric mean they reported in their main text).

SUPPLEMENTARY RESULTS

Following our characterization of the relation of optimal I/E and τ as a function of EPSG frequency in our standard model (Fig. 5b,c), we repeated the same analysis after varying other parameters of the model. Since these results were only briefly summarized in the main text, here we provide additional description of the results for each parameter.

Influence of variable EPSG and IPSG amplitude. As reported in the main text, addition of moderate variability in unitary EPSG and IPSG amplitudes following a normal distribution had almost no effect on optimal I/E and τ, whereas large variance following a log-normal distribution had a substantial effect at all but the lowest frequencies (but did not substantially alter the roughly linear relation to EPSG frequency) (Fig. 6). Although variability makes homeostasis more difficult to maintain, and thus increases MSR (Fig. 6d), it does not necessarily change optimal IPSG parameters substantially if the best those parameters can do is to counterbalance the average EPSG. In other words, IPSG can be understood as an expectation of EPSG amplitude, and increasing variability increases errors in prediction (and MSR) but may not alter the optimal expectation (for example, the mean).

With exception of figure 6, our simulations were simplified in using EPSG and IPSG of constant unitary amplitude. However, this simplification is not as unrealistic as it may initially appear. One source of variability results from presynaptic depression and facilitation of vesicle release. However, in contrast to typical conditions in brain slices, the amplitude of this variability has been found to be modest across a variety of neurons under natural conditions *in vivo* (Borst, 2010), including LGN (Carandini et al., 2007; Sincich et al., 2007; Weyand, 2007; Casti et al., 2008). With random timing of synaptic excitation at 40 Hz at the calyx of Held, the coefficient of variation (CV) of EPSC amplitude was only 8% (Hermann et al., 2007). The calyx of Held is a special case insofar as it consists of a single presynaptic fiber, and thus greater variance is to be expected in neurons with larger numbers of excitatory afferents.

Thus a second source of variability in real synaptic conductances at the cellular level results from variability in the number of synchronously active synapses (the extent of "spatial summation"). Our standard model was also simplified in this respect, effectively corresponding to a neuron with only one excitatory and one inhibitory synapse. However, EPSG could occur in each 1.0 ms time unit in our simulations. In a neuron with multiple excitatory inputs, synchronous excitation from two or more synapses within 1 ms may be quite rare. Thus if a neuron had 10 active excitatory synapses, each firing at 10 Hz, it may be a reasonable approximation to consider this to correspond to an EPSG rate of 100 Hz in our model with one synapse. It should also be noted that the EPSG that are of relevance to the present model and theory are the relatively small number of powerful "class 1 drivers" on soma or proximal dendrites and not the large number of weak "class 2" excitatory synapses on distal dendrites (Sherman, 2012), as discussed previously (Fiorillo et al., 2014).

Although these considerations suggest that our standard model without variance in unitary PSG is likely to be adequate for most purposes, we did take a step towards considering a neuron with multiple excitatory synapses when we sampled unitary PSG amplitudes from a log-normal distribution with high variance (Fig. 6a). A neuron with a large number of synapses would be expected to have a majority of its EPSG events caused by a small number of synchronously active synapses, and a minority of EPSG caused by larger numbers of coactive synapses. This would result in a skewed distribution, such as the log-normal distribution. Our log-normal distribution had a high variance (mean and standard deviation of 30 nS and 23.6 nS, CV of 0.79; the standard deviation corresponded to a factor of 2 from the mean, 15 to 60 nS). Thus it may be an appropriate model of a neuron with a large number of excitatory synapses. Although this variance more than doubled the optimal decay time at some frequencies (Fig. 6c), optimal decay times were still within the range of actual IPSG decay times (Fig. 10a). The dependence on EPSG frequency remained nearly linear (Fig. 6c).

Influence of mixed frequencies. Our use of EPSG patterns that are randomly distributed around a single mean is a crude approximation of more complex natural patterns. As a small step toward more realistic patterns, we considered a neuron that receives alternating periods in which EPSG occurred at 10 and 100 Hz (Supplementary Fig. 2a). When the amount of time at each rate was equal (0.1 s / cycle), optimal IPSG were nearly the same as with only the high rate of 100 Hz, even though the long-term average rate was cut to 55 Hz (Supplementary Fig. 2b). This is not surprising, since 10 times as many EPSG occurred during the high frequency period. However, when equal numbers of EPSG occurred during high and low frequency periods (0.1 s at 100 Hz alternating with 1.0 s at 10 Hz), corresponding to less than 10% of time at 100 Hz and a long term average EPSG rate of 18 Hz, optimal IPSG parameters were still closer to those that were optimal for a pure rate of 100 Hz than 10 Hz (Supplementary Fig. 2b). This is because short EPSG intervals contribute a disproportionate amount to MSR (Figs. 2b-d, 5c).

With IPSG optimized for each mixture, MSR was maximal when half of EPSG occurred at each frequency (10% of time at 100 Hz), indicating worse homeostasis for an equal mixture relative to unequal mixtures (Supplementary Fig. 2c). Although these intermediate IPSG parameters were the single best parameters for the long term given this equal mixture of EPSG at 10 and 100 Hz, they were not optimized for either 10 or 100 Hz. This points to the advantage of having a multiplicity of homeostatic mechanisms, beyond only IPSG with a single decay time constant, each being specialized for a distinct pattern of synaptic excitation.

Consideration of ‘mixed frequencies’ is important for comparison of our estimates of optimal IPSG to experimental observations (Fig. 10), since activity in some neurons is “sparse,” with long periods of inactivity punctuated by brief episodes of high-frequency excitation. For such neurons, the high-frequency periods will be of primary importance in determining optimal IPSG parameters, and the long-term average rate will be relatively inconsequential. We further address this issue with respect to figure 10 in Supplementary Methods.

Influence of action potentials. Simulations were performed with AP (Fig. 7) and without AP (Supplementary Fig. 1). We designed AP to be brief, as described in Online Methods. Optimal IPSG parameters with AP were similar to those observed without AP, although IPSG were somewhat larger and faster at most frequencies with AP (Fig. 7a, top and middle). Addition of AP introduced a non-linearity at high frequencies, with optimal τ being near 2 ms at 100 to 800 Hz. Addition of AP also reduced MSR at all but the lowest frequencies (Fig. 7a, bottom). At 800 Hz, MSR with AP was reduced to just 8% of that without AP. This was due to the brief reduction in excitability immediately following an AP, which naturally had greater influence at higher frequencies due to the prevalence of short inter-EPSG intervals. Since both positive and negative residuals were smaller with AP than without (Fig. 7a, inset at bottom), it appears that both sodium channel inactivation and potassium channel activation contributed to reducing MSR. By reducing MSR, AP promoted homeostatic excitability even in the presence of optimal IPSG.

Influence of membrane conductance. With higher membrane conductance, EPSPs will have earlier peaks and faster decay (due to a faster membrane time constant) (Fig. 7b, inset at top). The earlier peaks will reduce phasic inhibition, and the faster decay will reduce temporal summation. We tested two additional combinations of EPSG and G_L that were greater than our standard conductances but which resulted in the same unitary EPSP peak voltage in the absence of an IPSG (Fig. 7b and Supplementary Fig. 1a, inset at top) (like our standard E and G_L of 30 and 10 nS, respectively, combinations of 42 and 30 nS, and 120 and 200 nS, caused an EPSP evoked from -70 mV to reach a peak of -48 mV). These leak conductances of 10, 30 and 200 nS (100, 33, and 5 MΩ) resulted in membrane time constants of 24, 8, and 1.2 ms, respectively (in the absence of synaptic conductance).

At low frequencies, optimal IPSG decay time was faster with higher membrane conductance (Fig. 7b, middle), as one would expect given faster EPSP decay and less temporal summation. Whereas optimal I/E and τ were nearly monotonic functions across the full range of frequencies in our standard (low) conductance models, this was not the case for higher conductance. With the highest conductance, optimal I/E was maximal and τ minimal at 100 Hz, and optimal IPSG became smaller and slower at higher frequencies. This can be explained by a decrease in phasic inhibition at high frequencies. Spike latencies were earlier with higher conductance, and at higher frequencies (because membrane voltage usually remained near the threshold voltage at high frequencies, and the membrane time constant was fast; Supplementary Fig. 3a). At 800 Hz, half of all spikes in the intermediate conductance model, and all spikes in the high conductance model, had latencies of less than 1.0 ms after EPSG onset (as measured in simulations without AP, where a 'spike' was a crossing of -50 mV). With little or no contribution of phasic IPSG, optimization depended primarily or exclusively on the tonic component.

Our standard low conductance model was superior to the higher conductance models with respect to minimizing MSR (Fig. 7b, Supplementary Fig. 1a).

Influence of EPSG-IPSG delay. Inhibition has been found in most neurons to be delayed relative to excitation by 1 ms or more due to the need for feedforward transmission through one additional neuron. Despite the additional neuron, there are cases in which faster conduction via the inhibitory than excitatory pathway can result in inhibition preceding excitation (Lavallée et al., 2005; Roberts et al., 2013). In other neurons, excitation and inhibition may not derive from this "canonical feedforward circuit" (Zhou et al., 2010), and therefore a variety of temporal relationships can occur.

We tested E-I delays of -1.0, 0, 1.0 and 2.0 ms. E-I delay had little influence on optimal parameters at low frequencies. It had a stronger influence at higher frequencies, and that influence differed substantially between simulations with and without AP (compare Fig. 7c to Supplementary Fig. 1b). The E-I delay that best maintained homeostatic excitability (minimized MSR) also differed depending on presence of absence of AP, but the longest delay of 2.0 ms was clearly inferior in both cases, especially at high EPSG frequencies (Fig. 7c, Supplementary Fig. 1b).

Influence of chloride equilibrium potential. The chloride equilibrium potential (E_{Cl-}) varies over a large range across neuronal types and developmental stages (-40 to -90 mV). We tested E_{Cl-} of -60 to -100 mV (including our standard of -70 mV), which corresponds to a 5 fold range in driving force at spike threshold (-50 mV). In all simulations, we maintained the reversal potential of G_L at -70 mV

Optimal I/E and τ increased with E_{Cl-}, and thus optimal IPSG were both larger and longer lasting for E_{Cl-} of -60 mV (Fig. 7c). Optimal I/E should naturally increase with E_{Cl-} to compensate for the decrease in driving force. It is not so obvious why slower decay should be better with depolarized E_{Cl-}, but it may reduce MSR by keeping voltage near spike threshold. With E_{Cl-} of -100 mV, hyperpolarization was substantially greater between EPSP relative to E_{Cl-} of -60 mV, despite the fact that IPSG decay was faster (with optimal IPSG at each E_{Cl-}).

The influence of E_{Cl-} on homeostatic balance (MSR) was small compared to the influence of other parameters (compare bottom of Fig. 7d to 7a-c). MSR was minimized with E_{Cl-} of -60 or -70 mV at most frequencies in simulations with AP (Fig. 7d, bottom).

Spike probability depends on asymmetries in membrane properties. Optimal IPSG ensured that EPSPs were near threshold so that spikes and spike failures were both prevalent (Fig. 9a). However, the long-term spike probability (spikes/EPSG) depended on EPSG frequency as well as the criteria for selecting IPSG parameters. Learning rule 1 resulted in spike probabilities very close to 50% at all frequencies, whereas learning rule 2 resulted in probabilities near 40% at frequencies up to 100 Hz, and near 50% at 200 Hz and above. IPSG that minimized MSR resulted in spike probabilities that increased monotonically from 15% at 5 Hz to 42% at 800 Hz. Similar results were obtained in simulations without AP (Fig. 9a).

These differences in spike probability can be explained by considering the distributions of conductance, voltage, and residuals. At low EPSG frequencies, these distributions are highly skewed, with a mode at amplitudes below spike threshold and a 'tail' of higher amplitude events associated with spikes (Supplementary Fig. 3). As frequency increased, the distributions became more symmetric and their centers shifted closer to spike threshold. Qualitatively similar results were found for our log-normal distribution of unitary EPSG amplitudes (not shown). Spike-based rules (especially rule 1) tend to select IPSG parameters so that the median EPSP peak is near spike threshold, with the result that spike probability is close to 1/2. In contrast, minimizing MSR (distance from spike threshold) causes the mean EPSP peak to be near threshold. Because the distribution is skewed at low frequencies, this results in spike probability further below 1/2.

The central proposal of the theory tested here is that perfect homeostasis corresponds to the peak of every EPSP being exactly at spike threshold (Fiorillo et al., 2014). Although such perfection is not achievable, we previously reasoned that the long-term average spike probability should be near one half in neurons that are effective in maintaining homeostasis (Fiorillo et al., 2014). The present results demonstrate that the long-term spike probability predicted by theory to be present in neurons is not one half in general, but instead depends on the non-linear membrane properties and skewed distributions discussed above, which we did not consider previously. Since optimization of homeostatic conductances causes the mean or median excitatory event to be near spike threshold, a consequence of such skewed distributions (Supplementary Fig. 3) is a long-term spike probability below one half (Fig. 9a).

SUPPLEMENTARY METHODS

To compare our estimates of optimal IPSG parameters to experimental data (Fig. 10) required us to make many choices, especially concerning which types of neurons to include and their typical EPSG rates. Here we explain the rationale for our choices.

Theoretical aspects of estimating natural EPSG rates. We first describe our concept of 'EPSG frequency' in some detail, since it is not as familiar or simple as 'spike rate,' and because there is less data available that we can use to estimate it. Lack of information about EPSG rate was the reason that we limited our analysis to a relatively small subset of the many neurons in which τ has been measured.

Our working definition of EPSG frequency assumed synchronicity to be 1.0 ms, meaning that if EPSG at two excitatory synapses have onsets within less then 1.0 ms of one another, they will count as only one EPSG. This unit is satisfactory for most neurons, although it is too long for some fast neurons. Because of this issue, EPSG rate is easier to understood in neurons that receive a smaller number of excitatory synapses. EPSG rates will tend to be higher in neurons with large dendrites with many excitatory synapses. However, the EPSG rates of relevance here are only those from powerful "class 1" synapses that directly cause spikes, and not the very

large numbers of weak “class 2” synapses on the distal dendrites of cortical pyramidal neurons and thalamocortical neurons that do not directly cause spikes (Sherman, 2012). Furthermore, large dendrites necessarily slow the timescale of temporal integration, so that EPSG that are asynchronous in the dendrites may be effectively synchronous in the soma. The effective EPSG rate that matters here would be at the site of AP initiation if one considers the entire neuron, or at the location of a specific inhibitory synapse if one considers a learning rule based on local synaptic information. The effective rate at any specific location will necessarily be lower than the rate across all synapses, since summation of the effects of local EPSG across space necessarily takes time (“dendritic filtering”). These complexities are one reason for excluding cortical pyramidal neurons from our selected group of neurons used for measures of EPSG rate and τ (Supplementary Table 1).

Real EPSG are not randomly distributed around a single mean, and thus a single rate can only be an approximate description of the pattern of EPSG. For neurons in which EPSG are “sparse,” with long periods of low rates separated by brief periods of high rates, it is the high rates that are more relevant to our theory (Supplementary Fig. 2). This is because all EPSG intervals that are too long to exhibit temporal summation of EPSPs are functionally equivalent (for example, there is no difference between 0.1 and 10 s). Therefore the EPSG rate that is relevant to predicting IPSG properties using theory will be higher than a neuron’s average rate (assuming the interval distribution is not random). It also means that this rate is more difficult to estimate in neurons with sparse input, which includes most neurons in cerebral cortex.

In summary, the ideal neuron to which we would like to compare the predictions of our simple model would be electrotonically compact, have a single excitatory synapse, and a distribution of inter-EPSG interval that are not far from random (not sparse, or sinusoidal). It would also lack opponent IPSG (see Fig. 1c,d), since they could have different properties than the homeostatic IPSG of interest here (although in the only comparison we know, they had similar amplitude and decay time; Blitz and Regehr, 2005). Although these factors all pose a challenge, a greater challenge is to find adequate data on the statistics of natural EPSG patterns.

Selection of neurons. We selected 21 neurons for which we could obtain measures of both τ and typical EPSG input rate. We tried to include as many types of neuron as possible, but the number was limited by our ability to estimate EPSG rates. For reasons described above, we excluded cortical pyramidal neurons, as well as striatal medium spiny neurons. An additional difficulty with pyramidal neurons is that they receive a large number of distinct inhibitory inputs that differ in sub-cellular localization and can differ in τ. Their large dendrites also interfere with accurate voltage clamp and measurement of τ. However, we did include cerebellar Purkinje cells despite the problem of large dendrites (in contrast to cortical pyramidal neurons, Purkinje cells are more homogeneous, and have better characterized and more regular firing).

We excluded other types of neurons for which we could not accurately estimate EPSG frequencies. Olivocochlear neurons of the lateral superior olive (LSO) were excluded on this

basis, although they have been shown to have slower IPSG decay than principle neurons of the LSO (Sterenborg et al., 2010). The latter neurons were included since firing rates are known (see below).

Thalamocortical neurons in avian motor thalamus have large IPSG that decay rapidly (~2 ms) (Luo and Perkel, 2002), and they receive EPSG at high rates (~200 Hz) during singing (Goldberg and Fee, 2012). Thus they are consistent with our predictions. However, the large and fast IPSG that have been characterized in these neurons originate from basal ganglia and are "opponent" rather than "homeostatic," since their timing is anti-correlated with EPSG and spikes on a fine timescale (on slower timescales they are positively correlated, since both EPSG and opponent IPSG occur at higher rates during singing) (Goldberg and Fee, 2012).

Estimates of natural EPSG rates in the 21 selected neurons. Estimated EPSG rates are shown in Supplementary Table S1. These rates are meant to correspond to conditions of mild sensory stimulation, and to be biased slightly towards short EPSG intervals (higher rates) for reasons discussed above. We tried to be conservative in not deviating too much from experimental data, and thus we expect that we underestimated the effective rates that are relevant to our theory (since most reports give means, and EPSG and spike intervals are not randomly distributed; see above).

Among the selected neurons, EPSG rates have been recorded *in vivo* for thalamocortical neurons of LGN (Kaplan et al., 1987; Sincich et al., 2007; Weyand, 2007; Casti et al., 2008), cerebellar granule cells (Chadderton et al., 2004; Arenz et al., 2008), hippocampal dentate granule cells (Pernía-Andrade and Jonas, 2013; Kowalski et al., 2015), cochlear bushy cells (Englitz et al., 2009; Kuenzel et al., 2011), and neurons of medial superior olive (MSO) (van der Heijden et al., 2013). In these cases and others, EPSG input rates have typically been found to be approximately twice a neuron's firing rate, as summarized previously (Fiorillo et al., 2014). For neurons in which EPSG input rates have not been measured (other than the early auditory system; see below), the EPSG rate was estimated to be twice the firing rate observed with mild stimulation *in vivo*. EPSG rates for each type of neuron are discussed below.

Five of our neurons were from the early auditory system, for which good estimates can be made of the number of excitatory synapses. In order to avoid difficult choices about the precise experimental data to use for estimating rates, and to avoid logical inconsistencies across these connected neurons, we used estimates of the number excitatory presynaptic inputs to estimate typical EPSG input rates. We assumed that each auditory nerve (AN) fiber fires at 50 Hz (Louage et al., 2004), and that the spike output rate of each cochlear neuron was 1/2 of its EPSG input rate, as reported previously (Englitz et al., 2009; Kuenzel et al., 2011). From this, and the number of excitatory synapses, we estimated EPSG rates for each of the 5 types of neuron.

Spherical bushy cells (SBCs) of the ventral cochlear nucleus (VCN) typically receive excitation from 2 AN fibers, globular bushy cells (GBCs) receive 5, and T-stellate cells receive 7 (Cao and

Oertel, 2010). This accounts for our estimate of 100 and 350 Hz EPSG for SBCs and T-stellate cells (Supplementary Table 1), assuming independent firing of inputs (meaning that we did not account for the synchronous firing of presynaptic neurons within 1 ms; we expect that this assumption of independent firing introduces an error that is small relative to other sources of error). Our estimate of 100 Hz EPSG input rate for SBCs is consistent with experimental measurements (Kuenzel et al., 2011). A GBC receives 5 AN inputs (5 x 50 Hz = 250 Hz EPSG) and provides the sole input to a neuron in the medial nucleus of the trapezoid body (MNTB). Thus a neuron in MNTB would receive EPSG at 125 Hz. A neuron in MSO receives at least 4-8 excitatory fibers from SBCs (Couchman et al., 2010). Since this is probably a slight underestimate, we assumed 12 SBCs provide input to one MSO neuron, corresponding to 600 Hz. Although 12 inputs may be an overestimate, 600 Hz is probably an underestimate given that EPSP rates in MSO in the absence of acoustic stimuli are above 500 Hz (van der Heijden et al., 2013). Neurons in LSO also receive excitatory inputs from SBCs, and the number of inputs is likely to be similar (Case et al., 2011). Like MSO neurons, they fire at unusually high rates (Joris, 1996). Without such precise information as we have for MSO, we assumed that LSO principal neurons also receive EPSG at 600 Hz.

Neurons that use monoamine neurotransmitters, as well as cholinergic neurons in striatum, have median spontaneous rates across neurons of 2 or 3 Hz in behaving animals, with relatively little variability in inter-spike intervals (not sparse firing) (Bayer et al., 2007; Joshua et al., 2008; Fiorillo et al., 2013; Bouret and Richmond, 2015). Spikes in these neurons are internally generated ("spontaneous") in brain slices (in which synaptic transmission is absent), but it is not known whether any spikes are internally generated under natural conditions *in vivo*. According to the theory tested here, all spikes should be caused by EPSG (Fiorillo et al., 2014). The average EPSG rate in noradrenergic neurons of locus coeruleus in anesthetized rats was 3.7 Hz (Sugiyama et al., 2012). We estimated the firing rate under conditions of mild activation to be 5 Hz in each of these neuronal types, and therefore we assigned an EPSG rate of 10 Hz.

A slightly elevated rate in neurons of substantia nigra reticulata is about 100 Hz (Hikosaka and Wurtz, 1983), and therefore we assigned an EPSG rate of 200 Hz.

Thalamocortical neurons in LGN receive retinogeniculate EPSG at spontaneous rates of 15 or 20 Hz (Kaplan et al., 1987; Sincich et al., 2007; Weyand, 2007; Casti et al., 2008), and thus we estimated 40 Hz with mild activation. We assigned the same rate to thalamocortical neurons in primary auditory and somatosensory thalamus, which may fire at lower average rates but which might be expected to experience a higher incidence of very short EPSG intervals relative to visual neurons (Bartlett and Wang, 2011; Kwegyir-Afful et al., 2013). We estimated EPSG at 24 Hz in thalamic reticular neurons (TRN), based on a mean rate of 12 Hz in visual TRN neurons of awake mice (see Table S2 of Halassa et al., 2014).

Hippocampal dentate granule cells have exceptionally sparse activity, with very long periods of inactivity. Spontaneous EPSG occurred at an overall average rate of 15 Hz in awake and anesthetized rats, but most EPSG occurred in bursts with typical inter-EPSG intervals of about

25 ms (40 Hz) (Pernía-Andrade and Jonas, 2013; Kowalski et al., 2015). Virtually none of these EPSG caused spikes, even though neurons could fire up to at least 35 Hz in response to current injection (Pernía-Andrade and Jonas, 2013). Thus it is highly likely that dentate granule cells are driven at substantially higher EPSG rates on the rare occasions that their preferred stimuli occur. We therefore assigned them an EPSG rate of 50 Hz.

Hippocampal dentate basket cells inhibit granule cells (Bartos et al., 2001b). We presume that they resemble other fast-spiking, parvalbumin-positive interneurons found throughout much of the cerebral cortex, which are more broadly tuned than principle neurons and generally receive larger numbers of 'class 1' driving EPSG (Hu et al., 2014). As in other cortical areas, dentate basket cells are coupled to one another via gap junctions (Bartos et al., 2001b), which are similar in their effect to increasing the number of excitatory synapses and thus the EPSG rate. In CA1, parvalbumin-positive basket cells phase lock to 'sharp wave ripples' in the local field potential (LFP) of 200 Hz or more, and have population average firing rates of 75 to 122 Hz during these events (Lapray et al., 2012; Varga et al., 2012). In dentate granule neurons, IPSCs were coherent with the LFP at 75 to 100 Hz (Pernía-Andrade and Jonas, 2013), suggesting that local basket cells may spike at rates that high. These reported rates presumably correspond to stimulus-evoked rates (not average rates), and they are more likely to correspond to mild stimulation rather than maximal stimulation to the neuron's preferred stimulus. We assigned dentate basket cells an EPSG rate of 200 Hz, four times greater than dentate granule cells.

Cerebellar granule cells were found to have an average spontaneous EPSG rate of 4 Hz and rates of 75 Hz evoked by tactile stimulation (Chadderton et al., 2004). Similar EPSG rates were found with vestibular stimulation (Arenz et al., 2008). Granule cells have poorly characterized, multimodal receptive fields, and the evoked EPSG in these studies were mediated primarily by transmission at single synapses. Thus the maximal stimulus-evoked rates that have been reported are likely to be substantially lower than those that would occur with preferred stimuli that activate multiple excitatory synapses. We assigned an EPSG rate of 50 Hz.

Purkinje neurons and principle neurons of deep cerebellar nuclei (DCN) were assigned EPSG rates of 150 and 200 Hz based on estimated firing rates of 75 and 100 Hz, respectively (Rowland and Jaeger, 2005; Blenkinsop and Lang, 2011).

We are not aware of direct recordings of in vivo firing rates of nucleo-olivary neurons of DCN. They were found to fire spontaneously at 19 Hz in brain slices, with maximal driven rates near 70 Hz (in contrast to 400 Hz in principle neurons of DCN) (Najac and Raman, 2015). We assigned them an EPSG rate of 40 Hz.

Neurons of inferior olive were assigned an EPSG rate of 5 Hz based on spontaneous firing rates near 1 to 2 Hz (Chorev et al., 2007; Blenkinsop and Lang, 2011). They have sinusoidal subthreshold oscillations of 10 Hz related to electrical coupling (Chorev et al., 2007), suggesting a preponderance of inter-EPSG intervals near 100 ms. If that is the case, then regardless of the average EPSG rate, short inter-EPSG intervals (that cause temporal summation

of EPSP) may be more rare than if EPSG intervals were randomly distributed. Thus the effective EPSG rate (the rate that is comparable to the random intervals we tested) could be lower.

Experimentally measured IPSG decay times. Supplementary Table S1 shows EPSG rates and τ from 21 selected neurons. Since these were all from mammals, we assumed the natural temperature to be 37° and adjusted the reported values using a Q10 of 2.1 (both reported and adjusted values are shown). If decay was reported to have two or more exponential components, we used only the weighted time constant. For cell types in which we had more than one published report, we averaged across reports to obtain one weighted time constant per cell type.

$GABA_A$ IPSG decay times in cerebellar granule cells exhibit unusual variability, with a slow component that increases with presynaptic firing rate and may involve spillover (Crowley et al., 2009). This makes it difficult to choose a single value for τ. We chose a half-decay time of 7 ms based on spontaneous IPSCs, which appears to be approximately the median across cells (see Fig. 2J of Crowley et al., 2009). After adjustment for body temperature and conversion to a time constant, this resulted in τ of 7 ms.

Asynchronous release was observed at the synapse from nucleo-olivary neurons to inferior olive neurons, with the effective IPSG decay time depending on the presynaptic firing rate (Best and Regehr, 2009). Based on our estimate of nucleo-olivary firing (see above) (Najac and Raman, 2015), we estimated τ to be 60 ms at 20 Hz (see Fig. 4B of Best and Regehr, 2009), which we adjusted to 50 ms at 37°.

We did not find reports of τ for most neurons that use monoamines or acetylcholine as neurotransmitters. We wanted to include these neurons nonetheless, since they are known to have low and regular firing rates (see above). Therefore we estimated τ from figures of IPSCs by fitting a double exponential function and then finding the weighted time constant (Supplementary Table 1).

Experimentally measured IPSG to EPSG ratios (I/E). Fourteen values of I/E were obtained from 12 publications from 10 laboratories (Fig. 10c, Supplementary Table S2). Eleven of these were 'regular spiking' cortical neurons, and 8 were from primary auditory cortex. Rather than trying to get a single value for each type of neuron (as we did for τ, which involved averaging across multiple publications in some cases), we chose to use each reported I/E if it was distinct with respect to either neuronal type, cortical layer, species, or laboratory. Several publications from auditory cortex were excluded on this basis.

I/E should be measured *in vivo* so that excitatory and inhibitory presynaptic axons are coactived in their natural proportions. Thus we excluded reports obtained from brain slices, since electrical stimulation is likely to activate unnatural proportions of excitatory and inhibitory axons. The one exception was Blitz and Regehr (2005). Although their data was obtained from

brain slices with electrical stimulation of retinogeniculate axons, it is known that a thalamocortical neuron typically receive strong excitation from just one retinal neuron. Since the same retinal axon that causes a large EPSG in the thalamocortical neuron also drives feedforward IPSG via activation of dendrodendritic release of GABA from an interneuron, the natural proportions of IPSG to EPSG should be fairly well preserved in a brain slice. We estimated I/E from figure 3B of Blitz and Regehr (2005). It is worth noting that I/E was the same or slightly less for opponent ("non-locked") IPSG (08 vs. 1.1).

We excluded studies in which data on I/E was only available over extended periods of time (this includes Berg et al (2007) in spinal motoneurons and multiple studies in primary visual cortex). We ideally want to know peak conductance amplitudes within a few milliseconds after conductance onset. Because many in vivo studies align on the onset of external sensory stimuli (rather than EPSG onset), and then average over trials, our ideal measure of I/E is not available. The in vivo studies that we included generally measured initial peak synaptic conductances following sudden onset of sensory stimuli. In a study of primary visual cortex in awake mice, I/E was measured over the first 100 ms of responses following sudden stimulus onset (Haider et al., 2012). However, I/E did not change much over that time period (although IPSG decreased somewhat more than EPSG).

A second reason that it is important to measure I/E within milliseconds after conductance onsets is that opponent IPSG would be expected to contribute to inhibitory conductance over longer periods of time in some neurons, as illustrated in figure 1d. Opponent IPSG are anti-correlated with EPSG and spikes on a fine timescale, but can be positively correlated on slower timescales. In avian motor thalamus, long-term rates of EPSG and opponent IPSG both increase during singing (200-300 Hz), but because they are anti-correlated and suppression of spikes by opponent IPSG lasts only about 4 ms, corticothalamic neurons fire at about 100 Hz (Goldberg and Fee, 2012). Homeostatic IPSG should generally have shorter and less variable E-I delays compared to opponent IPSG, but this study demonstrates that the difference could be just a few milliseconds.

The only data we used in which we could not confirm a brief E-I delay was from cerebellar granule cells. Rates of synaptic excitation and inhibition both increased transiently following sensory stimulation, but the temporal relationship of EPSG and IPSG was not reported (Chadderton et al., 2004).

Another variable to consider is the relation of the sensory stimuli used in these studies to the neurons' excitatory and inhibitory receptive fields. For comparison to our theory and model, the stimulus would ideally be chosen to be the most common natural pattern, which would mean using broadband noise rather than a pure tone, or larger rather than smaller visual stimuli, to stimulate both the receptive field 'center' and 'surround.' However, excitatory and inhibitory receptive fields tend to be nearly the same, and all studies reported here used the same stimulus for evoking both excitation and inhibition. I/E did not depend strongly on stimulus location within the receptive field in some studies (Wehr and Zador, 2003), although it was greater when

the visual surround was activated without the center (Haider et al., 2013). We used values of I/E for stimuli near the receptive field center.

Supplementary References

Akerman CJ, Cline HT (2006) Depolarizing GABAergic conductances regulate the balance of excitation to inhibition in the developing retinotectal circuit in vivo. J Neurosci 26: 5117–5130.

Arenz A, Silver RA, Schaefer AT, Margrie TW (2008) The contribution of single synapses to sensory representation in vivo. Science 321: 977–980.

Awatramani GB, Turecek R, Trussell LO (2004) Inhibitory control at a synaptic relay. J Neurosci 24: 2643–2647.

Bartlett EL, Wang X (2011) Correlation of neural response properties with auditory thalamus subdivisions in the awake marmoset. J Neurophysiol 105: 2647–2667.

Bartos M, Vida I, Frotscher M, Geiger JR, Jonas P (2001) Rapid signaling at inhibitory synapses in a dentate gyrus interneuron network. J Neurosci 21: 2687–2698.

Bayer HM, Lau B, Glimcher PW (2007) Statistics of midbrain dopamine neuron spike trains in the awake primate. J Neurophysiol 98: 1428–1439.

Berg RW, Alaburda A, Hounsgaard J (2007) Balanced inhibition and excitation drive spike activity in spinal half-centers. Science 315: 390–393.

Best AR, Regehr WG (2009) Inhibitory regulation of electrically coupled neurons in the inferior olive is mediated by asynchronous release of GABA. Neuron 62: 555–565.

Blenkinsop TA, Lang EJ (2011) Synaptic action of the olivocerebellar system on cerebellar nuclear spike activity. J Neurosci 31: 14708–14720.

Blitz DM, Regehr WG (2005) Timing and specificity of feed-forward inhibition within the LGN. Neuron 45: 917–928.

Borst JGG (2010) The low synaptic release probability in vivo. Trends Neurosci 33: 259–266.

Bouret S, Richmond BJ (2015) Sensitivity of locus ceruleus neurons to reward value for goal-directed actions. J Neurosci 35: 4005–4014.

Bright DP, Aller MI, Brickley SG (2007) Synaptic release generates a tonic $GABA_A$ receptor-mediated conductance that modulates burst precision in thalamic relay neurons. J Neurosci 27: 2560–2569.

Bruzos-Cidon C, Llamosas N, Ugedo L, Torrecilla M (2015) Dysfunctional inhibitory mechanisms in locus coeruleus neurons of the Wistar Kyoto rat. Int J Neuropsychopharmacol 18: 1-11.

Cao XJ, Oertel D (2010) Auditory nerve fibers excite targets through synapses that vary in convergence, strength, and short-term plasticity. J Neurophysiol 104: 2308–2320.

Carandini M, Horton JC, Sincich LC (2007) Thalamic filtering of retinal spike trains by postsynaptic summation. J Vision 7: 20–20.

Case DT, Zhao X, Gillespie DC (2011) Functional refinement in the projection from ventral cochlear nucleus to lateral superior olive precedes hearing onset in rat. PLoS ONE 6: e20756.

Casti A, Hayot F, Xiao Y, Kaplan E (2008) A simple model of retina-LGN transmission. J Comput Neurosci 24: 235–252.

Chadderton P, Margrie TW, Häusser M (2004) Integration of quanta in cerebellar granule cells during sensory processing. Nature 428: 856–860.

Chorev E, Yarom Y, Lampl I (2007) Rhythmic episodes of subthreshold membrane potential oscillations in the rat inferior olive nuclei in vivo. J Neurosci 27: 5043–5052.

Couchman K, Grothe B, Felmy F (2010) Medial superior olivary neurons receive surprisingly few excitatory and inhibitory inputs with balanced strength and short-term dynamics. J Neurosci 30: 17111–17121.

Crowley JJ, Fioravante D, Regehr WG (2009) Dynamics of fast and slow inhibition from cerebellar Golgi cells allow flexible control of synaptic integration. Neuron 63: 843–853.

Dorrn AL, Yuan K, Barker AJ, Schreiner CE, Froemke RC (2010) Developmental sensory experience balances cortical excitation and inhibition. Nature 465: 932–936.

Englitz B, Tolnai S, Typlt M, Jost J, Rübsamen R (2009) Reliability of synaptic transmission at the synapses of Held in vivo under acoustic stimulation. PLoS ONE 4: e7014.

Fiorillo CD, Kim JK, Hong SZ (2014) The meaning of spikes from the neuron's point of view: predictive homeostasis generates the appearance of randomness. Front Comput Neurosci 8:49.

Fiorillo CD, Song MR, Yun SR (2013) Multiphasic temporal dynamics in responses of midbrain dopamine neurons to appetitive and aversive stimuli. J Neurosci 33: 4710–4725.

Froemke RC, Merzenich MM, Schreiner CE (2007) A synaptic memory trace for cortical receptive field plasticity. Nature 450: 425–429.

Goldberg JH, Fee MS (2012) A cortical motor nucleus drives the basal ganglia-recipient thalamus in singing birds. Nat Neurosci 15: 620–627.

Haider B, Häusser M, Carandini M (2013) Inhibition dominates sensory responses in the awake cortex. Nature 493: 97–100.

Halassa MM, Chen Z, Wimmer RD, Brunetti PM, Zhao S, Zikopoulos B, Wang F, Brown EN, Wilson MA (2014) State-dependent architecture of thalamic reticular subnetworks. Cell 158: 808–821.

Heiss JE, Katz Y, Ganmor E, Lampl I (2008) Shift in the balance between excitation and inhibition during sensory adaptation of S1 neurons. J Neurosci 28: 13320–13330.

Hermann J, Pecka M, Gersdorff von H, Grothe B, Klug A (2007) Synaptic transmission at the calyx of Held under in vivo-like activity levels. J Neurophysiol 98: 807–820.

Higley MJ, Contreras D (2006) Balanced excitation and inhibition determine spike timing during frequency adaptation. J Neurosci 26: 448–457.

Hikosaka O, Wurtz RH (1983) Visual and oculomotor functions of monkey substantia nigra pars reticulata. I. Relation of visual and auditory responses to saccades. J Neurophysiol 49: 1230–1253.

Houston CM, Bright DP, Sivilotti LG, Beato M, Smart TG (2009) Intracellular chloride ions regulate the time course of GABA-mediated inhibitory synaptic transmission. J Neurosci 29: 10416–10423.

Hu H, Gan J, Jonas P (2014) Fast-spiking, parvalbumin+ GABAergic interneurons: From cellular design to microcircuit function. Science 345: 1255263–1255263.

Huntsman MM, Huguenard JR (2000) Nucleus-specific differences in GABA(A)-receptor-mediated inhibition are enhanced during thalamic development. J Neurophysiol 83:350–358.

Jin X, Cui N, Zhong W, Jin XT, Jiang C (2013) GABAergic synaptic inputs of locus coeruleus neurons in wild-type and Mecp2-null mice. Am J Physiol Cell Physiol 304: C844–C857.

Joris PX (1996) Envelope coding in the lateral superior olive. II. Characteristic delays and comparison with responses in the medial superior olive. J Neurophysiol 76: 2137–2156.

Joshua M, Adler A, Mitelman R, Vaadia E, Bergman H (2008) Midbrain dopaminergic neurons and striatal cholinergic interneurons encode the difference between reward and aversive events at different epochs of probabilistic classical conditioning trials. J Neurosci 28: 11673–11684.

Kaplan E, Purpura K, Shapley RM (1987) Contrast affects the transmission of visual information through the mammalian lateral geniculate nucleus. J Physiol 391: 267–288.

Kirby LG, Freeman-Daniels E, Lemos JC, Nunan JD, Lamy C, Akanwa A, Beck SG (2008) Corticotropin-releasing factor increases GABA synaptic activity and induces inward current in 5-hydroxytryptamine dorsal raphe neurons. J Neurosci 28: 12927–12937.

Kowalski J, Gan J, Jonas P, Pernía-Andrade AJ (2015) Intrinsic membrane properties determine hippocampal differential firing pattern in vivo in anesthetized rats. Hippocampus 26: 668–682.

Kramer F, Griesemer DSE, Bakker D, Brill S, Franke J, Frotscher E, Friauf E (2014) Inhibitory glycinergic neurotransmission in the mammalian auditory brainstem upon prolonged stimulation: short-term plasticity and synaptic reliability. Front Neural Circuits 8:1400.

Kuenzel T, Borst JGG, van der Heijden M (2011) Factors controlling the input-output relationship of spherical bushy cells in the gerbil cochlear nucleus. J Neurosci 31: 4260–4273.

Kwegyir-Afful EE, Kyriazi HT, Simons DJ (2013) Weaker feedforward inhibition accounts for less pronounced thalamocortical response transformation in mouse vs. rat barrels. J Neurophysiol 110: 2378–2392.

Lapray D, Lasztoczi B, Lagler M, Viney TJ, Katona L, Valenti O, Hartwich K, Borhegyi Z, Somogyi P, Klausberger T (2012) Behavior-dependent specialization of identified hippocampal interneurons. Nat Neurosci 15: 1265–1271.

Lavallée P, Urbain N, Dufresne C, Bokor H, Acsády L, Deschênes M (2005) Feedforward inhibitory control of sensory information in higher-order thalamic nuclei. J Neurosci 25: 7489–7498.

Liu B-H, Li P, Sun YJ, Li Y-T, Zhang LI, Tao HW (2010) Intervening inhibition underlies simple-cell receptive field structure in visual cortex. Nat Neurosci 13: 89–96.

Louage DHG, van der Heijden M, Joris PX (2004) Temporal properties of responses to broadband noise in the auditory nerve. J Neurophysiol 91: 2051–2065.

Luo M, Perkel DJ (2002) Intrinsic and synaptic properties of neurons in an avian thalamic nucleus during song learning. J Neurophysiol 88: 1903–1914.

Magnusson AK, Kapfer C, Grothe B, Koch U (2005) Maturation of glycinergic inhibition in the gerbil medial superior olive after hearing onset. J Physiol 568: 497–512.

Matsui A, Jarvie BC, Robinson BG, Hentges ST, Williams JT (2014) Separate GABA afferents to dopamine neurons mediate acute action of opioids, development of tolerance, and expression of withdrawal. Neuron 82: 1346–1356.

Mayer F, Albrecht O, Dondzillo A, Klug A (2014) Glycinergic inhibition to the medial nucleus of the trapezoid body shows prominent facilitation and can sustain high levels of ongoing activity. J Neurophysiol 112: 2901–2915.

Miyazaki T, Lacey MG (1998) Presynaptic inhibition by dopamine of a discrete component of GABA release in rat substantia nigra pars reticulata. J Physiol 513: 805–817.

Momiyama T, Koga E (2001) Dopamine D2-like receptors selectively block N-type Ca2+ channels to reduce GABA release onto rat striatal cholinergic interneurones. J Physiol 533: 479–492.

Najac M, Raman IM (2015) Integration of Purkinje cell inhibition by cerebellar nucleo-olivary neurons. J Neurosci 35: 544–549.

Nugent FS, Penick EC, Kauer JA (2007) Opioids block long-term potentiation of inhibitory synapses. Nature 446: 1086–1090.

Pernía-Andrade AJ, Jonas P (2014) Theta-gamma-modulated synaptic currents in hippocampal granule cells in vivo define a mechanism for network oscillations. Neuron 81: 140–152.

Person AL, Raman IM (2012) Purkinje neuron synchrony elicits time-locked spiking in the cerebellar nuclei. Nature 481: 502–505.

Roberts MT, Seeman SC, Golding NL (2013) A mechanistic understanding of the role of feedforward inhibition in the mammalian sound localization circuitry. Neuron 78: 923–935.

Rowland NC, Jaeger D (2005) Coding of tactile response properties in the rat deep cerebellar nuclei. J Neurophysiol 94: 1236–1251.

Shen W, McKeown CR, Demas JA, Cline HT (2011) Inhibition to excitation ratio regulates visual system responses and behavior in vivo. J Neurophysiol 106: 2285–2302.

Sherman SM (2012) Thalamocortical interactions. Curr Op Neurobiol 22:575–579.

Sincich LC, Adams DL, Economides JR, Horton JC (2007) Transmission of spike trains at the retinogeniculate synapse. J Neurosci 27:2683–2692.

Smith AJ, Owens S, Forsythe ID (2000) Characterisation of inhibitory and excitatory postsynaptic currents of the rat medial superior olive. J Physiol 529: 681–698.

Sterenborg JC, Pilati N, Sheridan CJ, Uchitel OD, Forsythe ID, Barnes-Davies M (2010) Lateral olivocochlear (LOC) neurons of the mouse LSO receive excitatory and inhibitory synaptic inputs with slower kinetics than LSO principal neurons. Hearing Research 270: 119–126.

Sugiyama D, Hur SW, Pickering AE, Kase D, Kim SJ, Kawamata M, Imoto K, Furue H (2012) In vivo patch-clamp recording from locus coeruleus neurones in the rat brainstem. J Physiol 590: 2225–2231.

Sun YJ, Wu GK, Liu B-H, Li P, Zhou M, Xiao Z, Tao HW, Zhang LI (2010) Fine-tuning of pre-balanced excitation and inhibition during auditory cortical development. Nature 465: 927–931.

van der Heijden M, Lorteije JAM, Plauška A, Roberts MT, Golding NL, Borst JGG (2013) Directional hearing by linear summation of binaural inputs at the medial superior olive. Neuron 78: 936–948.

Varga C, Golshani P, Soltesz I (2012) Frequency-invariant temporal ordering of interneuronal discharges during hippocampal oscillations in awake mice. Proc Natl Acad Sci USA 109: E2726–E2734.

Venkataraman Y, Bartlett EL (2013) Postnatal development of synaptic properties of the GABAergic projection from the inferior colliculus to the auditory thalamus. J Neurophysiol 109: 2866–2882.

Vincent P, Armstrong CM, Marty A (1992) Inhibitory synaptic currents in rat cerebellar Purkinje cells: modulation by postsynaptic depolarization. J Physiol 456: 453–471.

Wanaverbecq N, Bodor AL, Bokor H, Slezia A, Luthi A, Acsady L (2008) Contrasting the functional properties of GABAergic axon terminals with single and multiple synapses in the thalamus. J Neurosci 28: 11848–11861.

Wehr M, Zador AM (2003) Balanced inhibition underlies tuning and sharpens spike timing in auditory cortex. Nature 426: 442–446.

Weyand TG (2007) Retinogeniculate transmission in wakefulness. J Neurophysiol 98: 769–785.

Wu SH, Kelly JB (1995) Inhibition in the superior olivary complex: pharmacological evidence from mouse brain slice. J Neurophysiol 73: 256–269.

Xie R, Manis PB (2013) Target-specific IPSC kinetics promote temporal processing in auditory parallel pathways. J Neurosci 33: 1598–1614.

Zhou M, Liang F, Xiong XR, Li L, Li H, Xiao Z, Tao HW, Zhang LI (2014) Scaling down of balanced excitation and inhibition by active behavioral states in auditory cortex. Nat Neurosci 17: 841–850.

Zhou Y, Liu B-H, Wu GK, Kim Y-J, Xiao Z, Tao HW, Zhang LI (2010) Preceding inhibition silences layer 6 neurons in auditory cortex. Neuron 65: 706–717.
